# Supplementary material for: Are Real-World Prosociality Programs Associated with Greater Psychological Well-Being in Primary School-Aged Children?
Source: Int J Environ Res Public Health. 2023 Mar 1;20(5):4403. doi: 10.3390/ijerph20054403 (PMC10002419; doi:10.3390/ijerph20054403)
Supplement: Supplementary file 1 [file ijerph-20-04403-s001.zip › ijerph-2209284-supplementary.pdf]

Supplemental Material: Are Real-World Prosociality Programs Associated with Greater Psychological Well-Being in Primary School-Aged Children?

In Study 2, participants in the *Dissimilar Recipient* condition could differ from the recipient of their C2C care package in varying ways. For example, participants could (1) differ in gender from the recipient, but be the same or similar age (i.e., within two years), (2) share gender, but be two or more years younger or older, or (3) differ by both gender and age. Thus, we pre-registered two different models to assess whether more nuanced operationalizations of dissimilarity may predict the change in pre- to -post volunteering happiness. Across each set of analyses, we provide the findings from our pre-registered regression models. In line with our directional predictions that volunteering to help a similar recipient would be more emotionally rewarding than helping a dissimilar recipient, we use one-tailed tests for all regression coefficients assessing similarity vs dissimilarity; all other tests were two-tailed. As recommended by Mundfrom and colleagues [92], we used Bonferroni corrections to address family wise error rate when testing the significance of regression coefficients within each model. Finally, as a more robust analytic strategy, we complement each regression model with a Repeated Measures ANOVA analysis.

In Model 1, we pre-registered to test whether gender differences and/or absolute differences in age may predict post-intervention happiness, while controlling for pre-intervention happiness. This model utilizes a more objective measure of differences in age by utilizing the absolute difference in age between the participant and recipient.We regressed post-intervention happiness on both differences in gender (0 = *gender mismatch*, 1 = *gender match*) and the absolute difference in age between participants and the recipients as well as pre-intervention happiness. As shown in Table S1, neither differences in gender nor absolute difference in age predicted post-intervention happiness using our one-tailed tests (ps > .703). Similarly, our repeated measures ANOVA revealed that the mean change in happiness did not differ as a

function of gender differences, $F(1, 213) = .38$, $p = .539$, $\eta_p^2 =.00$, 90%CI [.00, .02]. Meanwhile, the change in happiness from pre- to post-intervention did seem to differ as a function of absolute differences in age, $F(12, 213) = 2.22$, $p = .012$, $\eta_p^2 =.11$, 90%CI [.01, .13]. However, given the inconsistent results suggesting that packaging items for a child of a different age leads to greater happiness gains than for a child of similar age, we interpret this result with caution.

Table S1. Summary of OLS Regression Analyses (Study 2).

	Model 1					***Model 2***				
Predictor	*b* [CI]	*SE*	*ß* [CI]	*t*(234)	*p*	*b* [CI]	*SE*	*ß* [CI])	*t*(233)	*p*
**Pre-Intervention Happiness***	**.33<br>[.26, .41]**	**.04**	**.50<br>[.42, .58]**	**8.83**	**<.001**	**.33<br>[.26, .41]***	**.04**	**.50<br>[.42, .58]**	**8.80**	**<.001**
**Gender**<br>*[0 = Mismatch; 1 = Match]*	-.17<br>[-.30, -.04]	.08	-.12<br>[-.25, .01]	-2.16	.984	-.15<br>[-.28, -.02]	.08	-.11<br>[-.24, .02]	-1.93	.973
**Age**<br>*[Absolute Difference]*	.01<br>[-.01, .02]	.01	.03<br>[.01, .05]	.53	.703	-	-	-	-	-
**Age***<br>*[-1 = Younger; 0 = Same Age; 1 = Older]*	-	-	-	-	-	-.01<br>[-.10, .08]	.05	-.02<br>[-.12, .08]	-.29	.771
**Age**<br>*[0 = Same age; 1 = 1 year apart; 2 = 2+ years apart]*	-	-	-	-	-	.05<br>[-.02, .12]	.04	.08<br>[.01, .15]	.08	.881
R	.51					.52				
R^2	.26					.27				
Adjusted R^2	.25					.25				
ΔR^2	**.26**					**.27**				
F for ΔR^2	**$F(3, 234) = 27.72, p < .001$**					**$F(4, 233) = 21.10, p < .001$**				

Note. Significant regression coefficients and change in R^2 are in bold. Unless otherwise noted, all tests were one-tailed; * two-tailed test. For one-tailed tests in which the *t*-value was distributed opposite of the predicted direction, *p*-values were calculated as follows: 1 – ½ *p*-value. Bonferroni corrections were applied such that the *α* for each regression coefficient was divided by the number of predictors in the model.

Utilizing absolute differences in age is one method to assess dissimilarity in age. However, children do not often think of how similar or dissimilar they are to others in absolute terms. Rather, children may be much more apt to consider how they differ from the recipient based on whether they are younger or older. Moreover, children may consider being two or more years apart in age to be meaningfully different than being one year apart. Thus, in Model 2, we tested whether gender differences and/or subjective differences in age may predict post-intervention happiness, while controlling for pre-intervention happiness. Specifically, controlling for pre-intervention happiness, we regressed post-intervention happiness on (1) differences in gender between participants and recipients (0 = *gender mismatch*, 1 = *gender match*), (2) whether participants were the same age, older, or younger than the recipient (-1 = *younger*; 0 = *same age*, 1 = *older*), and (3) whether the participant and recipient were the same age, one year apart, or two or more years apart (0 = *same*, 1 = ± *one year*, 2 = ± *two or more years*). As in Model 1, regression analyses revealed that neither gender nor subjective age differences between the participant and the recipient predicted post-intervention happiness while controlling for pre-intervention happiness, βs < .05, ps > .771. Similarly, our repeated measures ANOVA revealed that neither differences in gender or subjective age differences predicted the change from pre- to post-intervention happiness, ps > .401. Taken together, we found no evidence that the change from pre- to post-intervention happiness was reliably predicted by dissimilarity in either gender or age and it did not matter whether we used more objective (i.e., absolute differences in age) or subjective operationalizations (i.e., being younger vs. older, being one vs. two or more years apart). Overall, these analyses suggest that helping a demographically similar recipient offers no greater emotional rewards than helping a dissimilar recipient.
